# Spatial helicity response metric to quantify particle size and turbidity of heterogeneous media through circular polarization imaging

**DOI:** 10.1038/s41598-023-29444-9

**Published:** 2023-02-08

**Authors:** Michael D. Singh, I. Alex Vitkin

**Affiliations:** 1 Department of Medical Biophysics, University of Toronto, Toronto, ON Canada; 2 Department of Radiation Oncology, University of Toronto, Toronto, ON Canada; 3 Division of Biophysics and Bioimaging, Princess Margaret Cancer Centre, Toronto, ON Canada

**Keywords:** Applied optics, Imaging

## Abstract

Backscattered circularly polarized light from turbid media consists of helicity-flipped and helicity-preserved photon sub-populations (i.e., photons of perpendicular and parallel circular handedness). Their intensities and spatial distributions are found to be acutely sensitive to average scatterer size and modestly sensitive to the scattering coefficient (medium turbidity) through an interplay of single and multiple scattering effects. Using a highly sensitive intensified-CCD camera, helicity-based images of backscattered light are captured, which, with the aid of corroborating Monte Carlo simulation images and statistics, enable (1) investigation of subsurface photonic pathways and (2) development of the novel ‘spatial helicity response’ metric to quantify average scatterer size and turbidity of tissue-like samples. An exciting potential application of this work is noninvasive early cancer detection since malignant tissues exhibit alterations in scatterer size (larger nuclei) and turbidity (increased cell density).

## Introduction

Bio-polarimetry remains arguably the least explored area of biophotonics due to the problematic effects caused by strong multiple scattering in thick mammalian tissues. These include (1) weak signals due to depolarization, and (2) intermixed polarization signals that are difficult to isolate due to the randomization of photon distributions^1^. Such obstacles, however, are slowly becoming more manageable as technologies and methodologies advance^2,3^, making way for novel or underexplored polarization effects to be exploited.

An interesting polarization effect that is largely unexplored is the *helicity* response of polarized light. Light’s helicity refers to the spin direction of its electric field vector which either traces a right- or left-handed corkscrew during propagation, and its response after scattering refers to the spin direction either becoming opposite to the incident state (i.e., flipped) or remaining the same (i.e., preserved)^4^. The sign (flipped $=$ negative and preserved $=$ positive) and magnitude of this response varies with detection angle^5–7^. General observations of helicity sign changes have been reported in several studies, typically regarding the sign-change dependency on average scatterer size: flipping is favoured for smaller sizes and preservation is favoured for larger sizes^4,8–13^. Further specific investigations of helicity effects are largely confined to numerical simulation results^5,11,14–17^. A complete experimental and theoretical (simulation) study of the helicity response would thus be desirable, to properly measure, understand, and quantify the sign, magnitude, and angular distribution of multiply-scattered circularly polarized light. This paper presents our research efforts in this important direction.

The helicity response may be useful for providing cancer diagnostics through its dependencies on important oncologic-related biophysical properties, including *scatterer size* and *turbidity*^17^. Cell nuclei are well known optical scatterers^18–20^ which increase in size during cancer progression (e.g., nuclear pleomorphism)^21^, enabling potential detection of cancers via higher measured average scatterer size^22–25^—a preferred alternative to invasive, subjective, and often costly biopsies^26,27^. Indeed, great interest in measuring and quantifying nuclear pleomorphism was initiated by the seminal studies from the Feld lab demonstrating the use of polarized light spectroscopy in human epithelia for early cancer detection^22,28–30^. Though it has been proposed that helicity changes may be useful for gleaning scatterer size information^4,11^, there remains to be a rigorous demonstration of this. It has also been observed that helicity flipping/preserving depends upon medium turbidity (quantified by the scattering coefficient)^12,13^—another scattering property whose alterations have been associated with malignancy^24,31,32^. Finally, circular polarization memory (i.e., ‘helicity survival’^4,8^) and ellipticity changes have shown importance for tumour detection^17,33–36^ and depth estimation^15^. Thus, there are various ways in which helicity effects may potentially prove useful for noninvasive cancer-related diagnostics.

Helicity-based signals may also offer insights into backscattering pathways (~ sampling depths) in turbid media such as tissues since helicity-flipped and helicity-preserved photon sub-populations likely follow different paths^7,14,37^. That is, helicity-flipped sub-populations will experience at least one large-angle scattering event (e.g., a retroreflection-like event) and hence in general penetrate shallower and emerge closer to the point of incidence. In contrast, the helicity-preserving sub-population will likely follow, on average, a deeper path of a larger number of near-forward scatterings and emerge further from the point of incidence. In the experimental and theoretical work that follows, we thus attempt to answer the following important questions: are these general suppositions true? Are such sub-populations measurable? How are they affected by medium properties? Does single scattering behaviour offer any insight into multiple scattering effects? Can the ensuing analysis be useful for biomedical tissue quantification? A better understanding of backscattered circularly polarized light dynamics may thus benefit biophotonics and related fields such as remote sensing where forward scattered (transmitted) light is unusable^38,39^, potentially facilitating the improvement of existing techniques and/or development of new ones.

We thus present a novel rigorous study of helicity-flipped and helicity-preserved images of backscattered circularly polarized light as a function of scatterer size and turbidity, enabling the development of the ‘spatial helicity response’ metric (SHRM) to quantify these two important medium properties. We corroborate the experimental images with Monte Carlo simulations and use the latter to further explore the underlying photon statistics including the average scattering angles and average numbers of scattering events experienced by flipped and preserved sub-populations. A highly sensitive intensified-CCD (ICCD) camera is employed to enhance the polarimetric images, which, along with the Monte Carlo simulation platform, exemplifies the use of enabling technologies and methodologies to aid the advancement of bio-polarimetry.

## Methods

### Turbid samples

Each suspension had a different combination of scatterer size and scattering coefficient. To achieve this, monodispersed polystyrene microspheres (Bangs Laboratories, Inc) of different diameters and concentrations were suspended in deionized water. Three sphere diameters were used: 0.20 μm, 0.58 μm, and 1.04 μm. The spheres had a refractive index of $n =$ 1.59 and the host medium (deionized water) had a refractive index of $n =$ 1.33. Concentrations were varied to yield scattering coefficients ranging from ${\upmu }_{s} =$ 50 cm^-1^ to ${\upmu }_{s} =$ 200 cm^-1^.

### Experimental system

**Figure 1:**
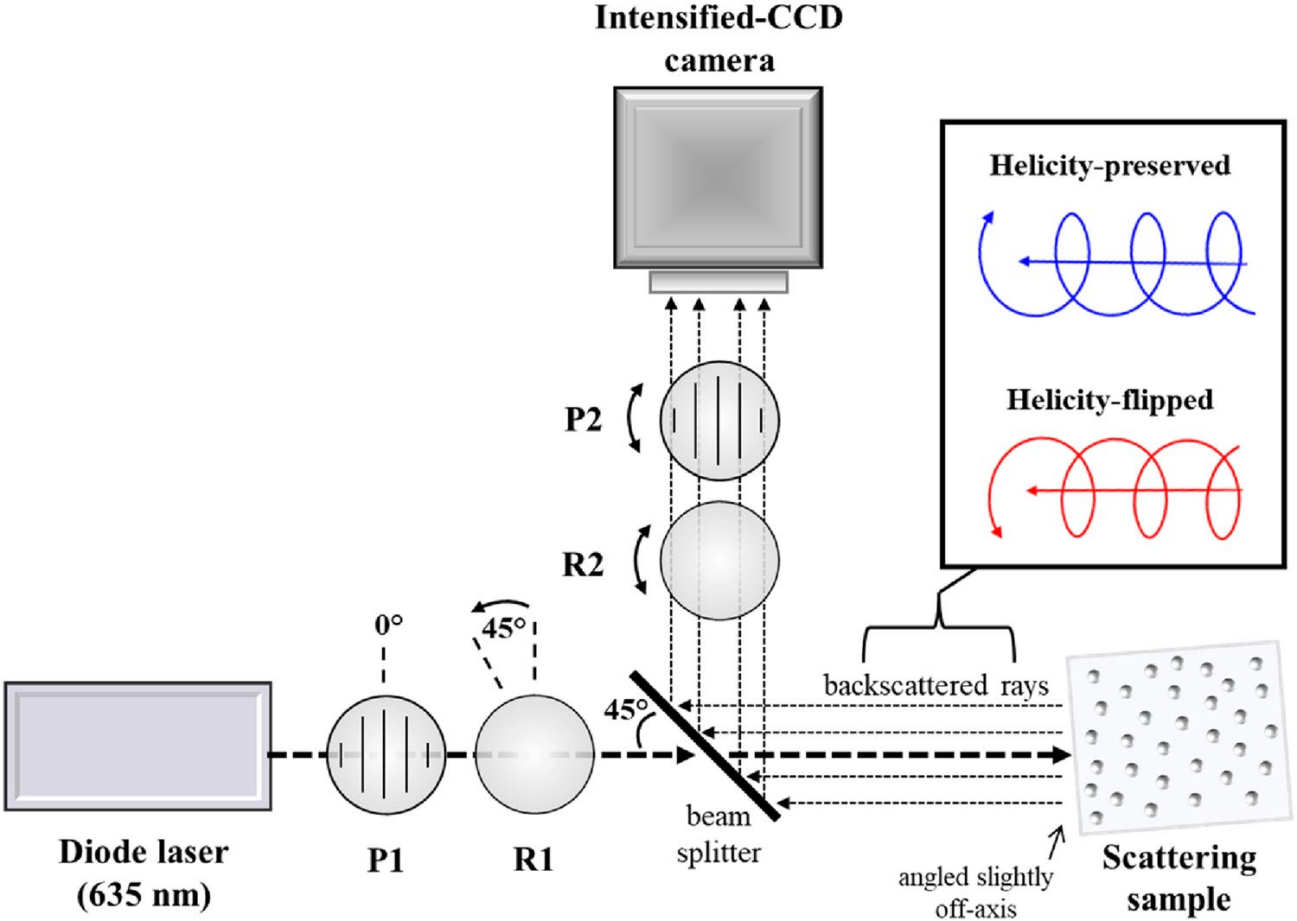
The schematic of the experimental system used to capture helicity-flipped and -preserved images of 180° backscattered circularly polarized light from polystyrene microsphere suspensions of varying scatterer size and scattering coefficients. A diode laser (λ = 635 nm) illuminated each sample, and an intensified-CCD (ICCD) camera imaged a fraction of the backscattered light reflected by a beam splitter. A linear polarizer (P1) and quarter wave retarder (R1) yielded incident left-circularly polarized light; a linear analyzer (P2) and quarter wave retarder (R1) transmitted right-circularly (flipped) and left-circularly (preserved) polarized components of the backscattered light. The sample was angled slightly off-axis to avoid detection of specular reflection.

Figure 1 shows the schematic of the experimental system which enabled the helicity studies, centered on the exact (180°) backscattering direction. The light source was a continuous-wave diode laser operating at λ $=$ 635 nm emitting a Gaussian beam with a full width at half maximum (FWHM) of approximately 3 mm, and the imaging device was an ICCD camera (PI-MAX® 3, Princeton Instruments). Left-circularly polarized light was generated by a linear polarizer (P1) followed by a quarter wave retarder (R1), which then passed through a beam splitter oriented at 45° with respect to the incident beam before illuminating each suspension. The suspensions were placed into a plastic cuvette of length 2.2 cm with a 2.2 × 2.2 cm silica optical window. The cuvettes were oriented a few degrees off-axis such that the specular reflection from the air-glass and glass-water interfaces avoided detection, leaving just the medium-backscattered light. A future approach can eliminate the silica window entirely and any of its potential effects by illuminating the sample from above (where there is no glass window), leaving just the air-sample interface. The suspensions backscattered the incident light to the beam splitter which reflected 5% of the intensity through a second quarter wave retarder (R2), linear analyzer (P2), and focusing lens (not shown) before reaching the ICCD camera. The resolution of the camera was 1024 × 1024 pixels, though only a 400 × 400 pixel region was used.

Although the beam splitter slightly complicated the polarization analyzer setup, the 180° backscatter detection geometry enabled symmetrical imaging of the light in the backward hemisphere. Moreover, the powerful sensitivity of the ICCD compensated for the intensity loss by the beam splitter. In the future, the ICCD camera can be triggered through its versatile electronic gating apparatus using dynamic polarization controllers such as photoelastic modulators^40^ and liquid–crystal variable retarders^41^ for rapid and automatic acquisition without mechanically moving parts.

Finally, for detailed helicity analysis, it is important to identify the illuminated region (IR) in each image, since the polarized light scattering dynamics inside and outside of it can differ as discussed in the following sections. To do this, we first imaged the beam by placing a glass plate at the position of the sample which reflected a fraction of the incident beam onto the camera and enabled determination of the beam’s width in pixels. The beams width was quantified by calculating the FWHM of the beam’s intensity profile.

## Theory

To better interpret the images of backscattered helicity-flipped and -preserved light, it is important to gain theoretical insight into the influence of scatterer size and turbidity. Single scattering theory for spheres has been rigorously laid out by Bohren and Huffman ^42^, as briefly summarized here. The angular distribution of scattered circular polarization intensity around a sphere can be calculated using its Mueller matrix multiplied by the incident Stokes vector:

1 $${\text{S}}_{{\text{s}}} = {\text{M}}_{{\text{S}}} \times {\text{S}}_{{{\text{in}}}}$$

2 $$\left[ {\begin{array}{*{20}c} {{\text{I}}_{{\text{s}}} } \\ {{\text{Q}}_{{\text{s}}} } \\ {\begin{array}{*{20}c} {{\text{U}}_{{\text{s}}} } \\ {{\text{V}}_{{\text{s}}} } \\ \end{array} } \\ \end{array} } \right] = \left[ {\begin{array}{*{20}c} {{\text{M}}_{{{11}}} \left( {\uptheta } \right){ }} \\ {{\text{M}}_{{{12}}} \left( {\uptheta } \right){ }} \\ {0 } \\ {0 } \\ \end{array} \begin{array}{*{20}c} {{\text{M}}_{{{12}}} \left( {\uptheta } \right){ }} \\ {{\text{M}}_{11} \left( {\uptheta } \right){ }} \\ {0 } \\ {0 } \\ \end{array} \begin{array}{*{20}c} 0 \\ 0 \\ {{\text{ M}}_{{{33}}} \left( {\uptheta } \right){ }} \\ { - {\text{M}}_{{{34}}} \left( {\uptheta } \right)} \\ \end{array} \begin{array}{*{20}c} { 0} \\ { 0} \\ {{\text{ M}}_{{{34}}} \left( {\uptheta } \right)} \\ {{\text{ M}}_{33} \left( {\uptheta } \right)} \\ \end{array} } \right] \times \left[ {\begin{array}{*{20}c} {{\text{I}}_{i} } \\ {{\text{Q}}_{i} } \\ {\begin{array}{*{20}c} {{\text{U}}_{i} } \\ {{\text{V}}_{i} } \\ \end{array} } \\ \end{array} } \right]$$

where ${\text{S}}_{{\text{s}}}$ and ${\text{S}}_{{{\text{in}}}}$ are the scattered and incident Stokes vectors, respectively, and ${\text{M}}_{{\text{S}}}$ represents the Mueller matrix of a single spherical scatterer; its elements being dependent on scattering angle ${\uptheta }$ (the angle between the incident and scattered light vector). The scattered circular polarization intensity can then be calculated as

3 $${\text{CP}}_{{\text{s}}} = \frac{{{\text{V}}_{{\text{s}}} }}{{{\text{I}}_{{\text{s}}} }} \times \frac{{{\text{V}}_{{\text{i}}} }}{{\left| {{\text{V}}_{{\text{i}}} } \right|}}{,}$$

where $\frac{{{\text{V}}_{{\text{s}}} }}{{{\text{I}}_{{\text{s}}} }}$ is the degree of circular polarization (DOCP, i.e., circular polarization memory). ${\text{V}}_{{\text{s}}}$ is calculated by subtracting the left-circular from the right-circular polarization intensities (yielding a signed value) and ${\text{I}}_{{\text{s}}}$ is calculated by adding those intensities. The sign of the scattered circular polarization intensity value is calculated using the second multiplication term of Eq. (3), which simply assigns a negative value if the scattered and incident V values have opposite signs to indicate a helicity flip, or a positive value to indicate helicity preservation if the signs are the same.

**Figure 2:**
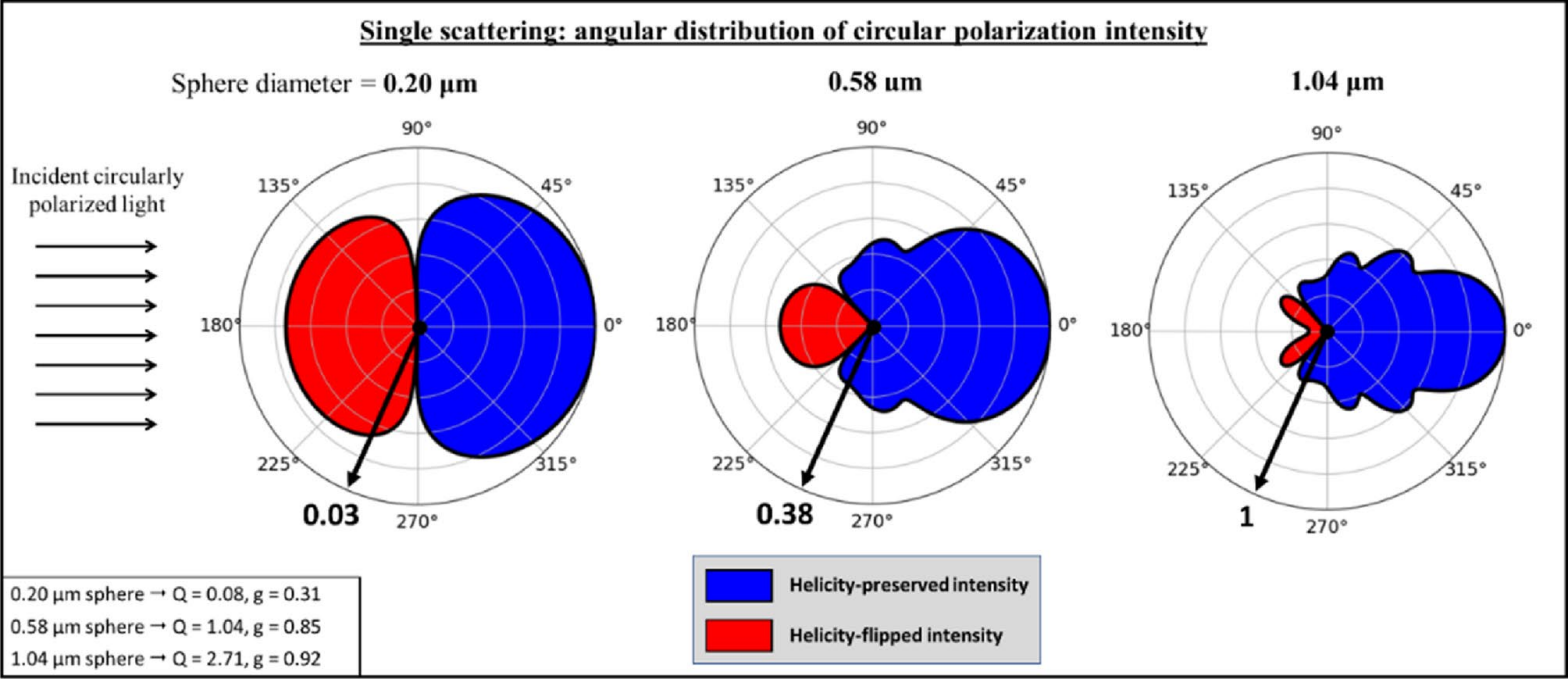
The sign and intensity of singly-scattered circularly polarized light depends on scattering angle and scatterer size, visualized on polar plots (log scale) for three sphere sizes, 0.20 μm, 0.58 μm, and 1.04 μm. The magnitude of each polar plot is scaled by the scattering efficiency of 1.04 μm spheres (Q $=$ 2.71), as indicated by the thick black arrows at 7 o’clock positions. The Mie parameters for the calculation were: light wavelength in vacuum ${\uplambda } =$ 635 nm, host medium refractive index $n_{m} =$ 1.33 (water), and scatterer refractive index $n_{s} =$ 1.59 (polystyrene). The calculated scattering efficiencies, Q, and scattering anisotropy factors, g, for each sphere diameter are shown in the bottom left. Helicity flipping occurs only in the backward direction, increasing in angular extent and in relative intensity as sphere size decreases.

Using the above equations, we compute the angular distributions of the degree of circular polarization for sphere diameters of 0.20 μm, 0.58 μm, and 1.04 μm, shown in Fig. 2a; the plots are colour-filled red to indicate helicity-flipping and blue to indicate helicity preservation. The incident light was circularly polarized and the following Mie parameters were kept constant: light wavelength in vacuum ${\uplambda } =$ 635 nm, host medium refractive index $n_{m} =$ 1.33 (water), and scatterer refractive index $n_{s} =$ 1.59 (polystyrene). The scattering efficiency, Q, and scattering anisotropy factor, g (‘g-factor’), for each sphere diameter is shown in the bottom left of the figure. (These are calculated for unpolarized incident light since they are referred to in later sections when examining multiply-scattered and hence largely depolarized photon packets. Future studies should examine polarization-sensitive Mie calculations in more detail). The magnitude of each polar plot is normalized by the scattering efficiency of 1.04 μm spheres (Q $=$ 2.71) to show how scattering intensity changes with sphere diameter. Arrows at the 7 o’clock positions indicate the relative sizes of the single scattering cross-section efficiencies for the three sphere sizes (for visual clarity, all are shown with the same diameter despite the ~ 30X difference in Q between large and small spheres).

As observed in Fig. 2, helicity flipping only occurs in the backward direction for these sphere diameters under these Mie parameters. In the context of single scattering, the ratio of flipping to preserving intensity increases as scatterer diameter decreases, because of more intense helicity-flipped scattering in the backward direction, but also due to the wider range of angles for which flipping occurs (e.g., the wide “red” lobe for the 0.20 μm spheres compared to the narrower double-lobe for the 1.04 μm spheres). Conversely, the absolute intensities of flipped and preserved beams both increase with scatterer size due to the higher overall scattering efficiency.

**Figure 3:**
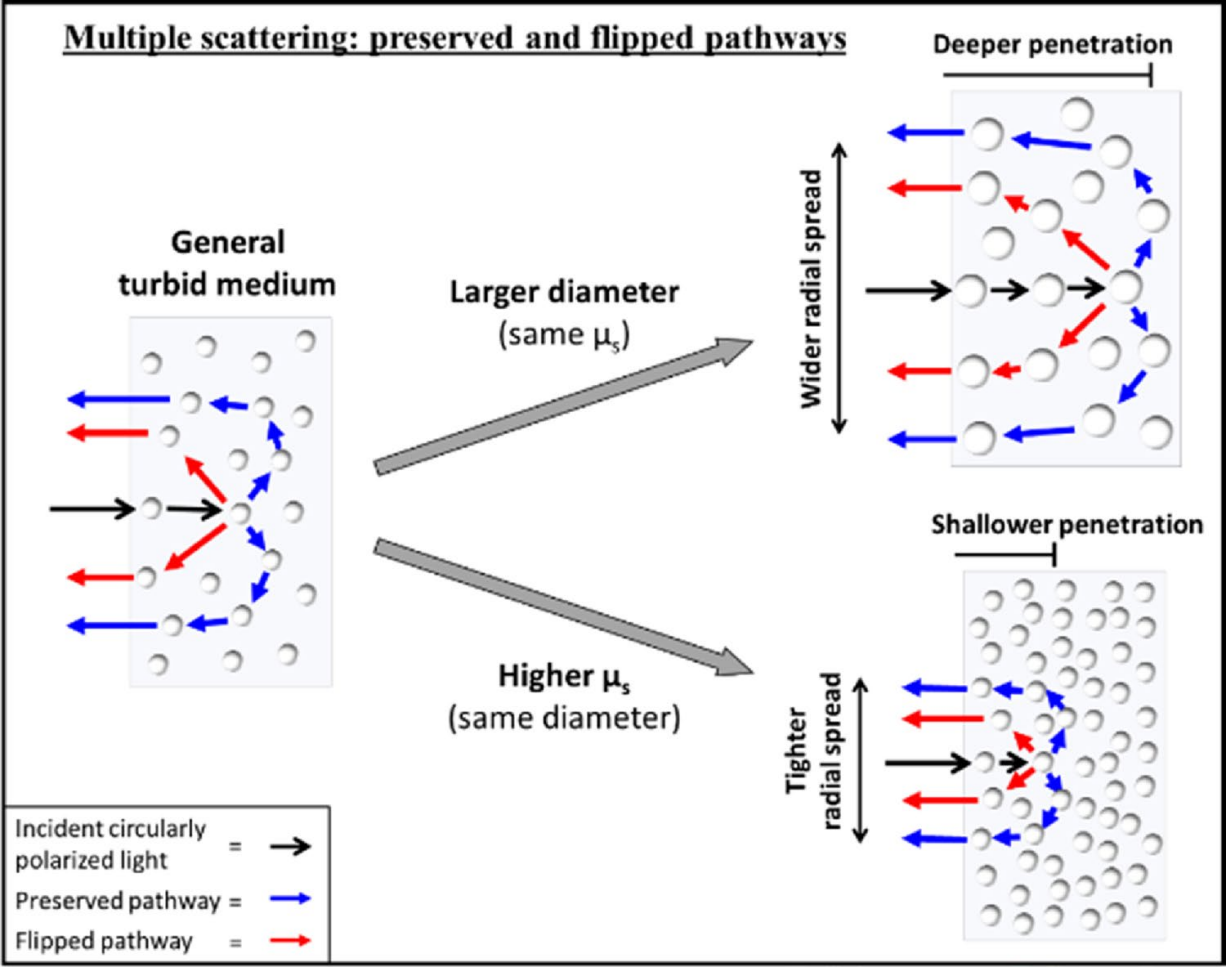
Schematic of flipped and preserved photons in multiple-scattering media, suggesting characteristic pathways distinguished by the presence (former) or absence (latter) of a large-angle backward-scattering event. As scatterer size increases (top right), one may expect deeper penetration and larger radial spread in the backward hemisphere. Higher turbidity (bottom right) is expected to confine light via the shorter mean-free path effect, resulting in shallower penetration and tighter radial spread. How these two properties influence the behaviour of both helicity-flipped and helicity-preserved subpopulations is discussed in the text.

Using the single-scattering plots in Fig. 2, we can theoretically reason how flipped and preserved photons may arise in backscattered light from a turbid medium (i.e., multiply scattered light). In general, helicity-flipped photons undergo at least one large-angle backward-scattering event, possibly preceded and followed by several forward-scattering events before re-emerging ^14^. In contrast, preserved light will mainly arise from photons which have taken a path that lacks a large-angle scattering (helicity-flipping) event; thus, the typical pathway will be more ‘arc-like’ with multiple small-angle forward-scattering events which eventually redirects into the backward hemisphere ^7,37^. Typical flipped and preserved pathways are depicted schematically in Fig. 3, along with general trends as sphere diameter and turbidity vary. We thus posit that the characteristics of flipped and preserved images will depend on (i) the angular distribution of single-scattering intensity for each sphere, which is directly linked to scatterer size (Fig. 2a), and (ii) the path assumed by the photons in the turbid media (Fig. 3), which is partly quantified by the mean-free path (MFP, the reciprocal of the scattering coefficient) ^12^. Thus, higher turbidity and hence shorter MFPs will result in more confined scattering and tighter backscattered images ^43^. Likewise, larger scatterers will enable deeper penetration (and likely more scattering) due to their higher probabilities of forward-scattering ^7,37^, causing photons to re-emerge at farther radial distances from the IR (Fig. 3). We thus expect the intensities to be spatially dependent ^17^. Furthermore, we expect the relative intensities of flipping and preserving to be mostly sensitive to scatterer size: for example, larger spheres will favour preserved light due to increased forward-scattering whereas smaller spheres may enhance helicity flipping. It is also important to note that helicity randomization (i.e., circular depolarization) will increase with the amount of scattering ^4^, as will likely occur at farther radial distances for example. In summary, the characteristics of helicity-flipped and helicity-preserved images of backscattered light will (likely) depend on scatterer size and (possibly) on turbidity, and can thus inform on these important properties.

## Results and discussion

### Experimental findings and model confirmation

**Figure 4:**
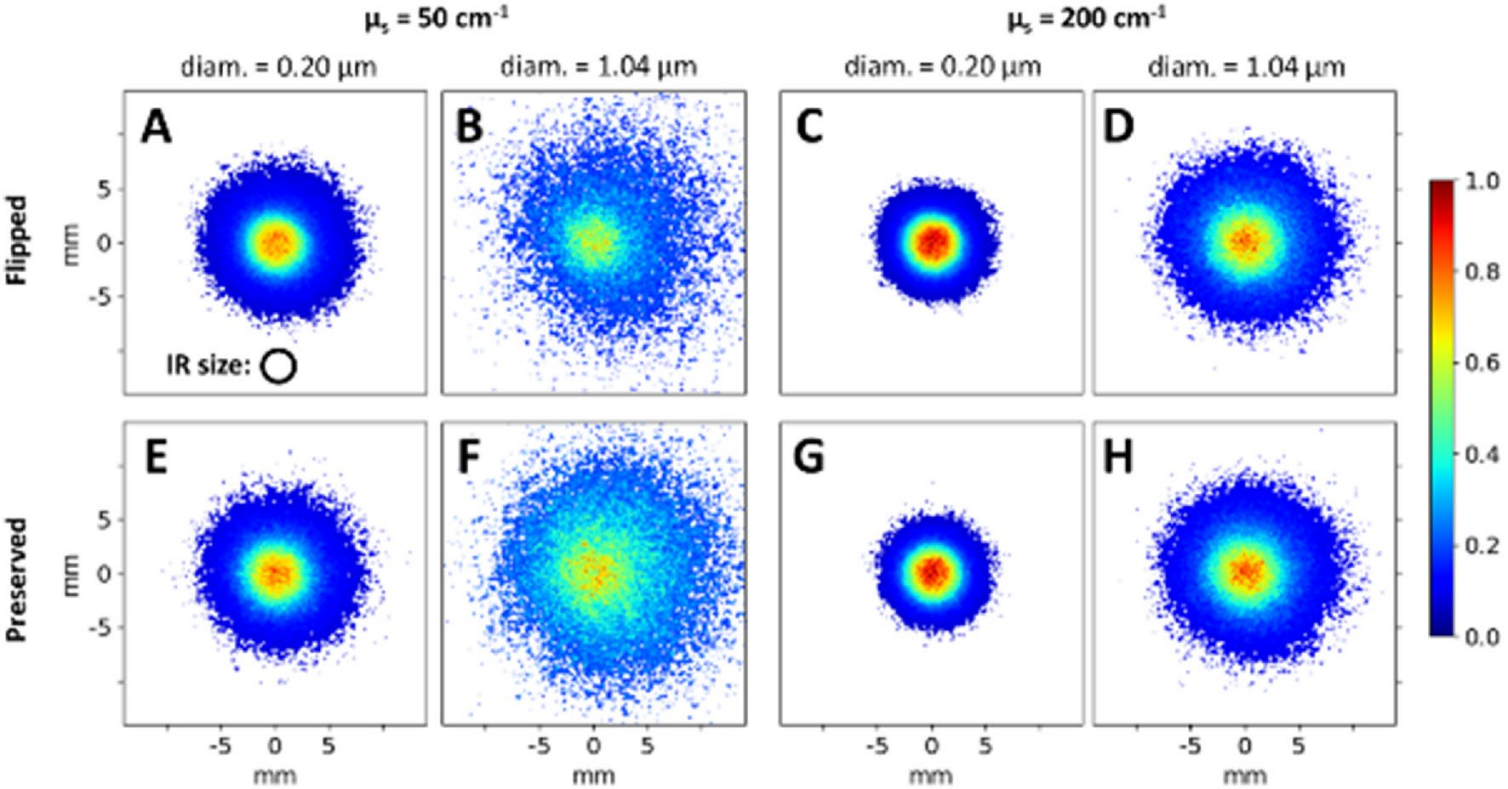
Helicity-flipped (top row) and helicity-preserved (bottom row) intensity images of backscattered circularly polarized light from four different monodispersed polystyrene suspensions, each having a sphere diameter of either 0.20 μm or 1.04 μm and scattering coefficient of either 50 cm^−1^ and 200 cm^−1^ (see labels). The drawn circle in A shows the size of the centrally illuminated region. It is visually apparent that the characteristics of resultant images are dependent on the scatterer size and turbidity (for discussion of noted trends, see text).

Figure 4 shows the flipped and preserved experimental images for suspensions of 0.20 μm and 1.04 μm spheres at scattering coefficients of ${\upmu }_{s} =$ 50 cm^−1^ and 200 cm^−1^. The complete set of experimental images including other sphere diameters and turbidity values can be found in Fig. S1 of the Supplementary Section. Upon qualitative examination, it is evident that the images are indeed sensitive to sphere diameter and turbidity. We note the following trends: (1) The intensity distributions are tighter for the smaller spheres and appear more peaked in the IR (compare any left–right image pair). (2) The intensity distributions shrink with turbidity (compare A to C, B to D, E to G, and F to H). (3) The helicity-preserving distributions appear to be wider than the flipped ones at lower turbidity (e.g., compare B to F), whereas higher turbidity appears to equalize the distribution widths. The Monte Carlo simulated versions of these images in Fig. S2 of the Supplementary Section also exhibit these general characteristics; such agreement between theory and experiment lends additional credence to these results. We try to quantify, understand, and explain these and other less visually obvious trends below.

**Figure 5:**
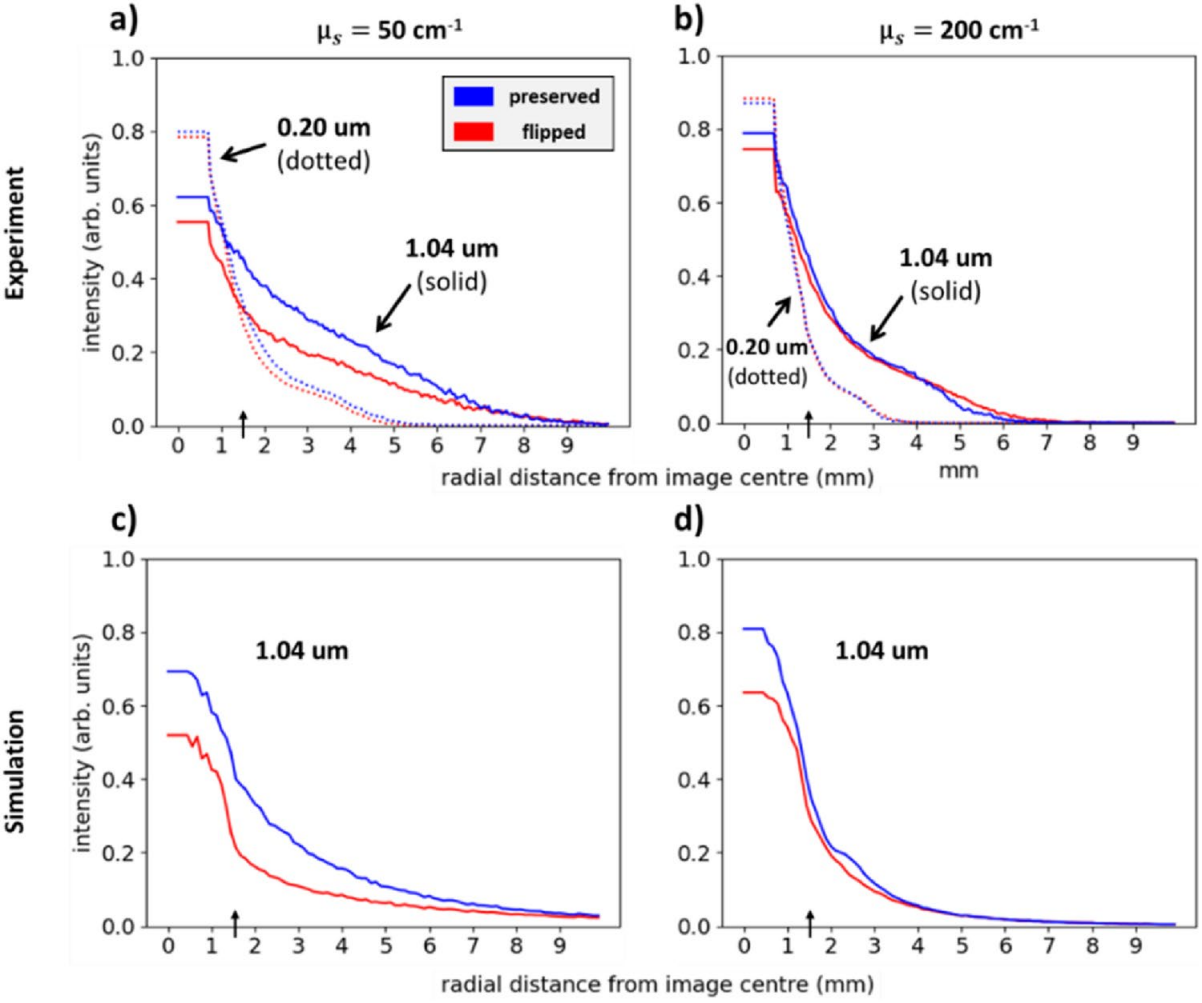
Helicity-flipped and helicity-preserved radial profiles of images corresponding to suspensions with scattering coefficients of 50 cm^−1^ and 200 cm^−1^ and sphere diameters of 0.20 μm (dotted lines) and 1.04 μm (solid lines). The vertical arrows along the x-axes mark the outer edge of the IR. The simulation radial profiles in (**c**) and (**d**) exclude the 0.20 μm data (see text for details). Scatterer size and turbidity information can be gleaned by analyzing these profiles, for example, enhanced preserving-to-flipping ratio by larger spheres and increased confinement (narrowing of the profiles) with turbidity.

**Figure 6:**
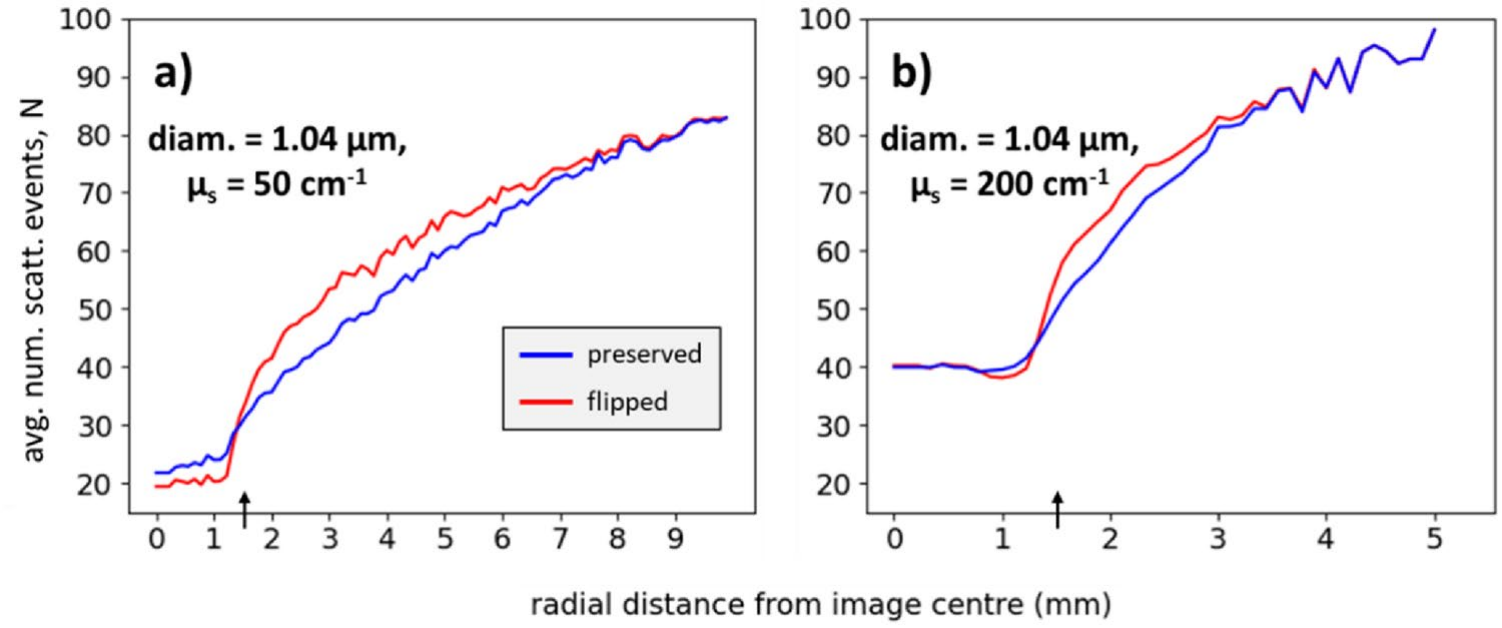
The average number of scattering events, N, for flipped and preserved photons as a function of radial distance from the centre of the simulation images of suspensions with 1.04 μm spheres at ${\upmu }_{s} =$ 50 cm^−1^ (left) and 200 cm^-1^ (right). There is an interesting reversal at the IR boundary whereby flipped photons switch from undergoing less to more scattering than preserved photons; see text for discussion. The vertical arrows mark the outer edge of the IR.

The above observed characteristics can be more quantitatively examined by analyzing the central radial profiles of each image. Figure 5a and b show the flipped (red) and preserved (blue) radial profiles from the images above, for sphere diameters 0.20 μm (dashed) and 1.04 μm (solid) and the two scattering coefficients (a) ${\upmu }_{s} =$ 50 cm^−1^ and (b) ${\upmu }_{s} =$ 200 cm^-1^. Note that the central 15 pixels of each image are averaged for the radial profile computation to decrease signal choppiness, resulting in the horizontal lines from R $=$ 0 mm to ~ 0.8 mm. Clearly, (1) the intensity distributional spread outside the IR decreases with sphere diameter, attributable to the shallower penetration and hence closer re-emergence of photons to the IR. Within the IR, the intensities are more peaked for the smaller scatterers. The higher flipping in the IR is due to the higher backward-scattering of smaller spheres (essentially increased reflectance). The higher preservation in the IR is less obvious, but likely occurs because of the higher chance of ‘far-forward’ scattering (e.g., 45°–90° angles) for smaller spheres (see Fig. 2) which enables more preserved photons to take shorter arc-length pathways and return within the IR. (2) Higher turbidity exerts a confinement effect on the light whereby the radial spreads are decreased due to the shallower penetration (see Fig. 3) and higher peak intensity in the IR due to the shorter MFP which enables more photons to re-emerge within the IR. (3) At ${\upmu }_{s} =$ 50 cm^−1^ the preserved curves are wider than the flipped one: for example, notice the preserved intensity for 1.04 μm spheres reaches the 20% level at ~ 5 mm whereas the flipped intensity reaches that level at ~ 3 mm. This is due to the more arc-like pathway of preserved photons that re-emerge at farther distances from the IR (see Fig. 3). This effect is less prominent at ${\upmu }_{s} =$ 200 cm^−1^, likely because of the associated increased scattering which depolarizes the light at rather short radial distances such that the curves become indistinguishable (see Fig. 6 and its associated discussion). An additional trend that was not easily observed in the images of Fig. 4 is the greater preserved$-$flipped difference for larger spheres (especially at lower turbidity, see Fig. 5a), further demonstrating that larger scatterers enhance helicity-preserved light via increased forward-scattering^14,44^.

It is observed that the flipped and preserved simulation profiles in Fig. 5c and d converge at different radial distances for the two examined turbidities: at ~ 9 mm for ${\upmu }_{s} =$ 50 cm^−1^ and ${\text{at}}\sim 4\;{\text{mm}}\quad {\text{for}}\;{\upmu }_{s} =$ 200 cm^−1^ (experimental panels (a) and (b) above also exhibit this, although these are harder to quantify, especially at the higher scattering coefficient). Such homogenization of intensities of the two sub-populations is an indication of total circular depolarization, whereby the helicities have been randomized enough such that there is an equal chance of flipping and preserving^8^. This convergence behaviour stems from the relationship between radial distance and the number of scattering events, the latter of which governs the statistics of circular depolarization ^4^, as will be discussed in the following section.

Importantly, the radial profiles shown in Fig. 5c and d from the Monte-Carlo-simulated images of the 1.04 μm sphere suspensions exhibit strong agreement with, and hence validate the corresponding experimental findings. It must be noted that the simulation—experiment agreement for smaller spheres does show slight (~ 5–15%) discrepancy, especially at lower turbidity (as identified in our previous publication^12^); the cause is currently being investigated. In the meantime, for brevity and visual clarity the 0.20 μm simulation results are not shown, and we focus on the 1.04 μm simulation data for further insight.

### A deeper dive into polarization statistics (modeling studies)

Before proceeding to a potential practical application of the presented polarization helicity formalism (Section "A practical polarization metric deliverable"), we examine the details of subsurface propagation statistics for the flipped and preserved photons as afforded by the Monte Carlo simulation results. Though there have been previous studies on the behaviour of these photon types using numerical simulations^5,11,14–16^, to the best of our knowledge this section represents the first such simulation-based investigation accompanied by corroborating experimental data to reinforce the validity of the results. Figure 6 displays a comparison of the simulation-obtained average number of scattering events N as a function of radial distance for the 1.04 μm spheres at ${\upmu }_{s} =$ 50 cm^-1^ (a) and ${\upmu }_{s} =$ 200 cm^-1^ (b). As expected, N increases with radial distance due to the longer resultant total pathlengths^45^. Moreover, N is clearly higher for ${\upmu }_{s} =$ 200 cm^-1^ such that circular depolarization occurs at shorter radial distances. Since the radial distance of re-emerging photons is a function of N and hence penetration depth, it may be exploited to *tune* the polarimetric depth sensitivity (akin to the work of the da Silva lab^46–49^). Furthermore, the above observations suggest that the ‘convergence distance’ (radial extent beyond which the two sub-populations become indistinguishable) may be a useful measure of turbidity. However, sphere size must also be taken into account since it influences the required amount of scattering for circular depolarization^4^; thus, consideration of additional variables may be wise, such as the preserved-to-flipped ratio in the IR to inform on scatterer size.

Figure 6 shows that preserved photons undergo more scattering in the IR than do flipped photons (especially evident in the ${\upmu }_{s} =$ 50 cm^−1^ results). This is due to the multiple small-angle events required for preserved photons to be backward-redirected, compared to the one large-angle redirection event encountered by flipped light. Unexpectedly though, N is higher for flipped photons outside the IR, which remains puzzling and is simply noted for now; however, it may be the cause of the weaker flipped-to-preserved intensities outside the IR in Fig. 5 (i.e., due to higher depolarization of flipped photons). Finally, the profiles converge at ~ 80 scattering events for both turbidities, suggesting this is when ~ complete circular depolarization occurs for this sphere size and refractive index (size parameter X = 6.8, refractive index mismatch M = 1.20); this agrees well with the theoretical calculation shown in Fig. 1 of Ref.^4^ for the same Mie parameters. (Also, as a ‘reality check’, notice that the curves in Fig. 6 converge at the same points as the corresponding curves in Fig. 5c and d (~ 9 mm and ~ 4 mm)).

**Figure 7:**
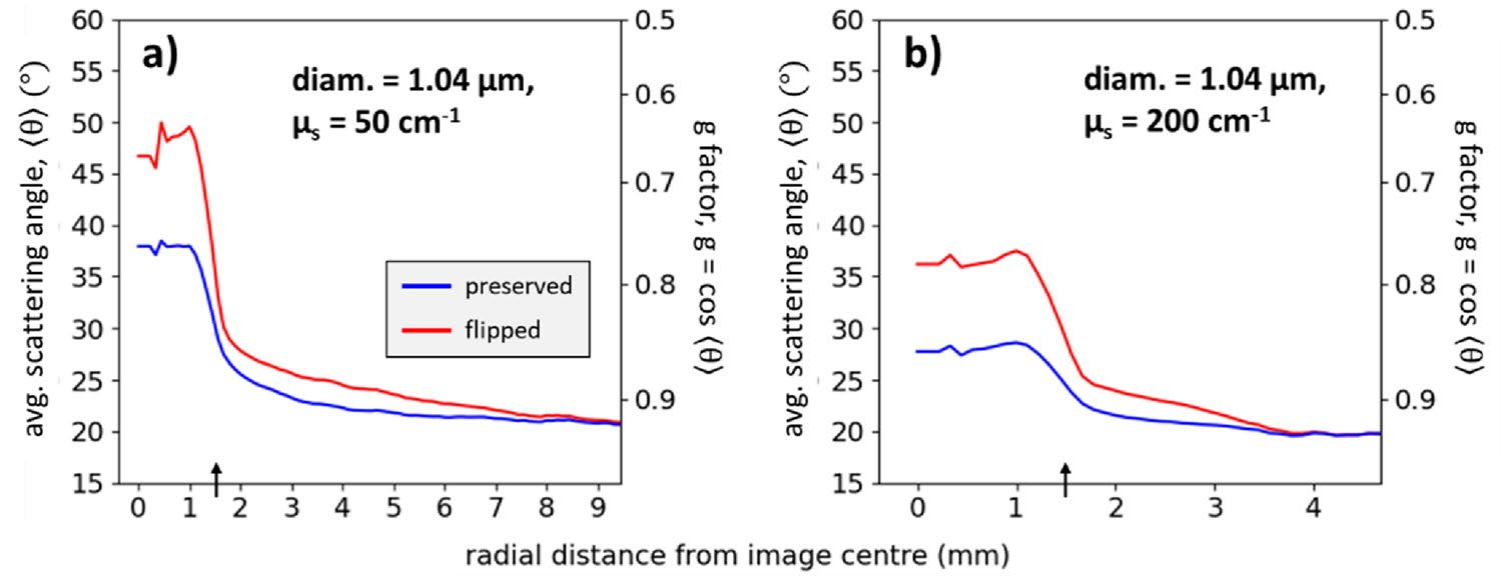
The average scattering angle for flipped and preserved photons as a function of radial distance for suspensions with 1.04 μm spheres at ${\upmu }_{s} =$ 200 cm^-1^. The flipped profiles are higher than the preserved profiles due to the large angle scattering events of flipped photons.

In addition to scattering event counts, we can also gain insight into the subsurface photon paths through analysis of the average scattering angle, ${\uptheta }$. Figure 7 shows ${\uptheta }$ as a function of radial distance for 1.04 μm spheres at (a) ${\upmu }_{s} =$ 50 cm^-1^ and (b) ${\upmu }_{s} =$ 200 cm^−1^; both panels also show the scattering anisotropy ‘g-factor’ (calculated as g $=$ cos ${\langle \uptheta \rangle }$) on the right y-axes. The average scattering angle of the flipped and preserved photons are most different in the IR, then gradually converge to the state of total depolarization. Importantly, the convergences in both plots occur at g $\approx$ 0.92 which is the theoretically calculated g-factor of these spheres for unpolarized light (see bottom left of Fig. 2), adding credence to these results. The preserved photons for ${\upmu }_{s} =$ 50 cm^−1^ yield a g-factor of ~ 0.75 in the IR, which is lower than 0.92 because of the somewhat larger angles required to redirect the photons into the backward hemisphere (e.g., see Fig. 3); the preserved g-factor is higher in the IR at ${\upmu }_{s} =$ 200 cm^-1^ (lower average scattering angle) due to the increased scattering. One may expect the flipped photons to have its ${\uptheta }$ closer to 135° in the IR—the angle threshold at which flipping occurs for these spheres (see Fig. 2)—however, the ${\uptheta }$ values are much lower, indicating that most flipped photons are forward-scattered several times before and/or after the large-angle flipping event, contrary to some previous thinking^5,44^.

To better understand the average scattering angle behaviour of flipped photons in the IR, let us take a closer look at the ${\upmu }_{s} =$ 50 cm^−1^ data. The flipped g-factor in the IR of ~ 0.65° must arise from a combination of photons which undergo some number of forward-scattering events along with at least one large-angle scattering event of $>$ 135°. As an illustrative example of how such a g-factor may possibly arise, consider two types of pathways: (1) 19 near-forward scatterings along with one direct backward-scatter event to flip the helicity, and (2) photons which are singly backward-scattered by the first few layers of scatterers. Suppose that 90% of photons are type (1) and 10% are type (2), yielding

4 $${\text{g}} = \frac{{(19 {\text{\,events }} \times 0.92 + 1{\text{ event}} \times {\text{ cos}}\left( {180^\circ } \right)}}{{20 {\text{\,events }}}} \times 90\% + \frac{{1{\text{ event}} \times \cos \left( {180^\circ } \right)}}{{1 {\text{\,event}}}} \times 10\% = 0.64$$

approximately the simulated result of g ~ 0.65. In Eq. (4), g $=$ 0.92 was used as the near-forward-scattering g-factor and $\cos \left( {180^\circ } \right)$ was used as the direct backward-scatter event. The numbers in this sample calculation were arbitrarily chosen to simply illustrate the computation of the flipped g-factor. A future version of the Monte-Carlo simulation platform should track the photon population distribution with respect to scattering event counts (i.e., a histogram) to investigate the actual values of these g-factor calculations. Clearly there is much more to learn and interpret from these subsurface helicity simulation studies, particularly in regard to backscattering dynamics which is of key importance in bio-polarimetry. For example, knowledge that a measurable contribution of the flipped signal arises from scattering in the first few microns of a sample may enable superficial polarimetric sampling of tissues (where many pathologies arise such as tooth decay^50^ and skin cancers^51^). Moreover, further understanding of these propagation pathways may prove fruitful in imaging applications for improved contrast in turbid media^38,39,52,53^.

### A practical polarization metric deliverable

**Figure 8:**
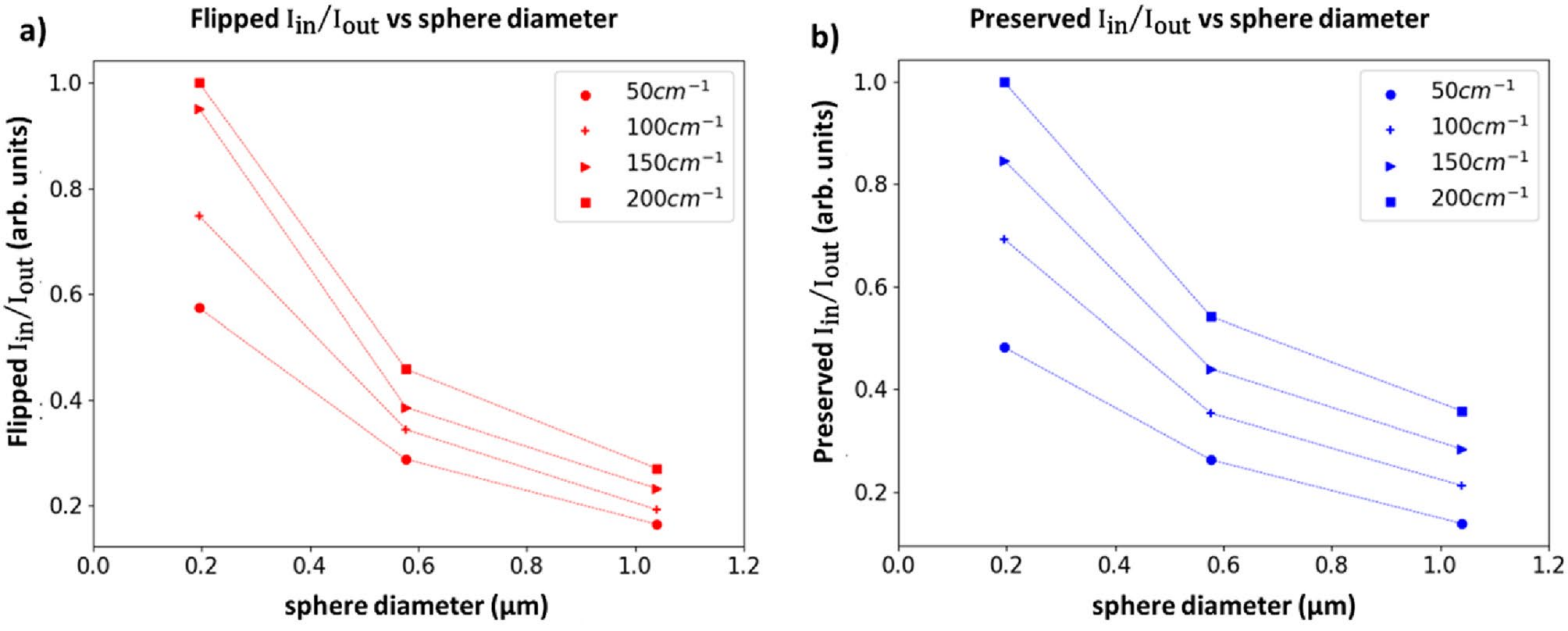
Intensity ratios inside/outside the IR for (**a**) flipped and (**b**) preserved photon subpopulations, for three different sphere diameters and four different turbidities. These ratios enable appreciable separation of data, however, ambiguity remains, necessitating incorporation of additional metrics to achieve uniqueness (for details, see text). The data are normalized by the 0.20 μm, ${\upmu }_{s} =$ 200 cm^−1^ values. Symbols represent experimental results; the lines are a guide for the eye.

As an illustrative example of how this analysis may be used in practice, we explore the capability of helicity-flipped and -preserved images to inform on scatterer size and turbidity. Considering again Fig. 5 and its associated discussion, we now make use of the following profile features that correlate with size and turbidity: (i) the total flipped and preserved intensities within the IR, ${\text{I}}_{{{\text{in}}}}$; (ii) the total of these intensities outside the IR, ${\text{I}}_{{{\text{out}}}}$; and (iii) the radial spread of these intensities outside the IR, ${\text{R}}_{{{\text{out}}}}$. Thus, there are six quantities, $\left\{ {{\text{I}}_{{{\text{in}}}} , {\text{I}}_{{{\text{out}}}} ,{\text{R}}_{{{\text{out}}}} } \right\}_{{{\text{flipped}}}}$ and $\left\{ {{\text{I}}_{{{\text{in}}}} , {\text{I}}_{{{\text{out}}}} ,{\text{R}}_{{{\text{out}}}} } \right\}_{{{\text{preserved}}}}$. ${\text{R}}_{{{\text{out}}}}$ is the distance from R $=$ 3 mm to the R value at which the corresponding intensity (flipped or preserved) reaches zero. ${\text{I}}_{{{\text{in}}}}$ and ${\text{I}}_{{{\text{out}}}}$ are calculated by summing the flipped or preserved intensity profiles from R $=$ 0 mm to R $=$ 3 mm and by summing over the ${\text{R}}_{{{\text{out}}}}$ region, respectively. Larger scatterers yield lower ${\text{I}}_{{{\text{in}}}}$, higher ${\text{I}}_{{{\text{out}}}}$, and higher ${\text{R}}_{{{\text{out}}}}$. Higher turbidity resulted in higher ${\text{I}}_{{{\text{in}}}}$, lower ${\text{I}}_{{{\text{out}}}}$, and lower ${\text{R}}_{{{\text{out}}}}$. These trends apply for both flipped and preserved quantities. The flipped and preserved ratios ${\text{I}}_{{{\text{in}}}} /{\text{I}}_{{{\text{out}}}}$ will thus be inversely proportional to scatterer size and proportional to turbidity, as is indeed seen in Fig. 8. These ratios enable appreciable separation by size and turbidity, but fail to achieve ‘uniqueness’ and hence some ambiguity remains (e.g., the flipped ratios for 0.58 μm, ${\upmu }_{s} =$ 50 cm^-1^ and 1.04 μm, ${\upmu }_{s} =$ 200 cm^-1^ overlap).

We find that unique separation can be achieved by multiplying those ratios by their corresponding R_out_ values, and then multiplying them together to yield a potentially useful helicity-based sample characterization metric:

5 $${\text{SHRM}} = \left\{ {{\text{I}}_{{{\text{in}}}} /{\text{I}}_{{{\text{out}}}} \times {\text{R}}_{{{\text{out}}}} } \right\}_{{{\text{flipped}}}} \times \left\{ {{\text{I}}_{{{\text{in}}}} /{\text{I}}_{{{\text{out}}}} \times {\text{R}}_{{{\text{out}}}} } \right\}_{{{\text{preserved}}}}$$

**Figure 9:**
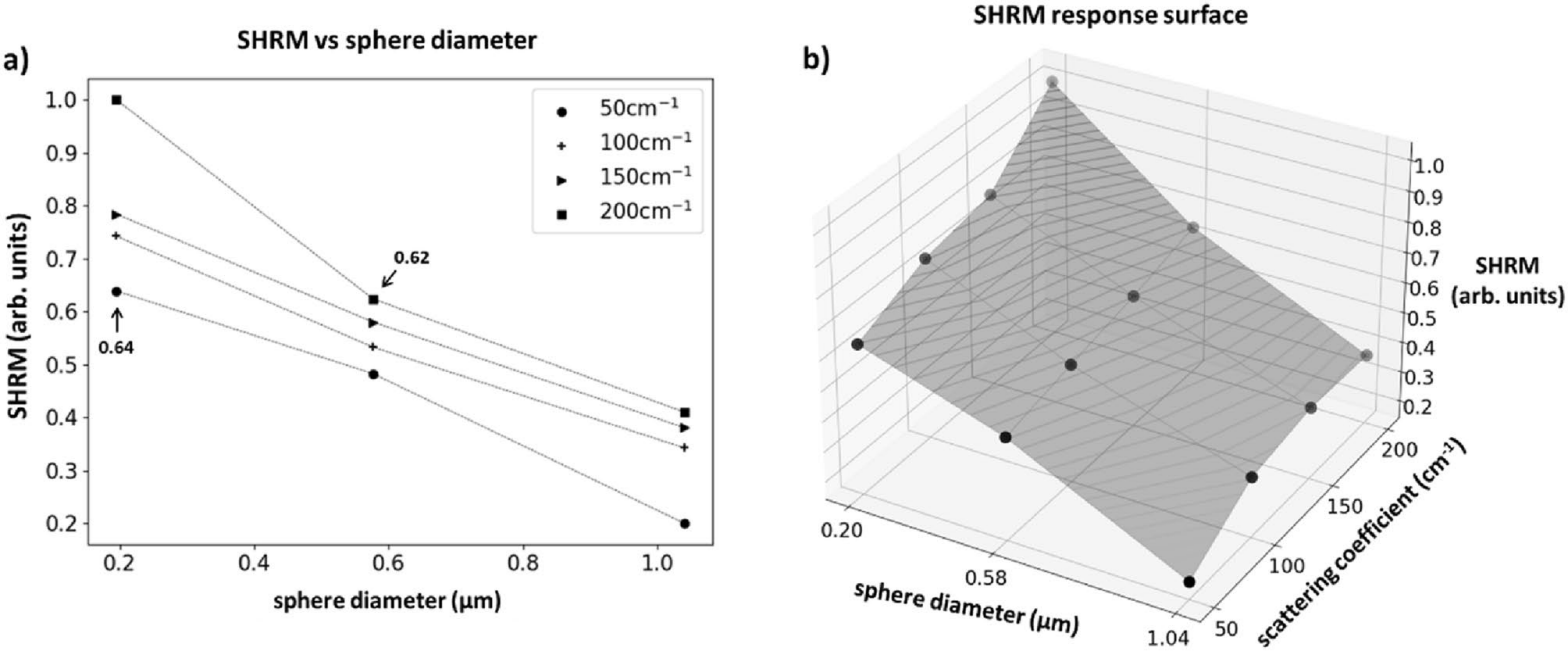
The composite ‘spatial helicity response’ metric (SHRM) is highly sensitive to sphere diameter with essentially linear dependence, and modestly sensitive to turbidity. The SHRM is plotted as (**a**) a 2D scatter plot and (**b**) a 3D response surface against scatterer diameter and scattering coefficient. The SHRM enables simultaneous quantification of scatterer size and turbidity, except at lower values of ${\upmu }_{s}$ where this unique association becomes ambiguous (arrows in (**a**); for details, see text). The data are normalized by the 0.20 μm, ${\upmu }_{s} =$ 200 cm^−1^ SHRM value. Symbols represent experimental results; the lines are a guide for the eye.

This composite product, which we term the ‘spatial helicity response’ metric (SHRM), is highly sensitive to sphere diameter and modestly sensitive to turbidity. This is shown as a 2D scatter plot in Fig. 9a, and as a 3D response surface in Fig. 9b. As seen, it is encouraging that a given SHRM value seems to correspond to a unique combination of scatterer size and scattering coefficient. This may then offer a helicity-analysis-based practical experimental means to determine these important medium properties. Yet this uniqueness does seem to weaken at the lower range of the examined turbidity values. For example, SHRM at ${\upmu }_{s} =$ 50 cm^−1^ for the smallest spheres almost overlaps with SHRM of midsized spheres at highest turbidity (arrows in Fig. 9a) and similarly for the midsized spheres at ${\upmu }_{s} =$ 50 cm^−1^ and largest spheres at ${\upmu }_{s} =$ 200 cm^-1^; thus in these cases, SHRM-based discrimination remains slightly ambiguous. Fortunately, most mammalian tissues are highly turbid in the visible/near-IR spectral range with scattering coefficients ${\upmu }_{s} >$ 100 cm^-1^^54^, so this shortcoming of the SHRM may be less biomedically relevant. (Interestingly, if the ${\upmu }_{s} =$ 50 cm^-1^ data are removed, the flipped ${\text{I}}_{{{\text{in}}}} /{\text{I}}_{{{\text{out}}}}$ can alone achieve unique separation in Fig. 8). Thus, the scatterer size and ${\upmu }_{s}$ of an unknown turbid sample can be simultaneously quantified by acquiring helicity-flipped and helicity-preserved images, calculating the SHRM, and plotting it onto a response surface whereby its location can be mapped to those medium properties.

The SHRM can potentially prove useful for diagnostic applications concerning particle size and turbidity such as cancer detection^17,33–36^. Toward such applications though, this approach requires further investigation in more tissue-like media with broad scatterer size and refractive index distributions as opposed to the simpler suspensions of essentially singular scatterer size and two-phase refractive index (polystyrene and water) implemented in this study. The impact of refractive index may become interesting since it influences helicity flipping/preserving angles and the g-factor (scattering anisotropy)^6^; however, it is likely less important in the context of signal sensitivity to nuclear pleomorphism during cancer progression since the refractive index of nuclei is estimated to increase by less than 5%^55^ whereas their size can more than double^28^. Additionally, illumination wavelength must often be selected based on the absorption profile of the target tissue; thus, it will be important to gain insight into the effects of wavelength on the helicity-based images through its influence on scattering dynamics (e.g., MFP, g-factor, penetration depth, etc.).

A key advantage of the SHRM is that it offers simultaneous scatterer size and turbidity information through measurement of just circular polarization rather than also including two linear polarization orientations (Q and U) as typically required for Stokes polarimetry. This practical experimental simplicity is further apparent when compared to the far more complex Mueller matrix polarimetry, which typically requires sixteen combinations of incident and scattered Stokes vectors measurements. To further simplify the approach, as noted earlier, only the flipped intensity may possibly be measured in order to provide sufficiently useful diagnostic information. Other additional useful helicity metrics may be discovered with further analysis (for example the ‘convergence distance’ or the preserved-to-flipped intensity ratio in Fig. 5b), however that will be pursued in a separate study. Finally, backscattered images can also be obtained using incident linearly polarized light to enable comparison of linear and circular polarization responses for additional information and enhanced medium diagnostics^12,34,56^.

## Conclusion

In this work we analyze helicity-based images of circularly polarized light backscattered from polystyrene microsphere suspensions of varying scatterer size and turbidity through consideration of single and multiple scattering effects, both experimentally and by simulation. The strong agreement between theory and measured data was encouraging in validating the entire framework, and in enabling a more detailed study of helicity-flipped and helicity-preserved backscattering pathways via simulation-obtained statistics. The potential practical utility of the helicity-based imaging was suggested through the introduction of the ‘spatial helicity response’ metric (SHRM) which enabled simultaneous quantification of scatterer size and turbidity. This represents the first demonstration of direct helicity analysis to inform on medium properties. Excitingly, this novel experiment exemplifies the importance of advanced technology for bio-polarimetry applications: the intensified-CCD camera greatly facilitated exact-backscatter (180°) detection by enhancing the extremely faint beam-split polarization signals. Additionally, the ICCD camera can be used in tandem with dynamic no-mechanically-moving-parts polarization modulators to gain practical SNR and measurement speed advantages to enable rapid, robust, and automatic image acquisition^40,41^.

## Supplementary Information

Supplementary Information.

## Data Availability

The datasets generated during and/or analysed during the current study are available from the corresponding author on reasonable request.
