# Supplementary material for: Spatial helicity response metric to quantify particle size and turbidity of heterogeneous media through circular polarization imaging
Source: Sci Rep. 2023 Feb 8;13:2231. doi: 10.1038/s41598-023-29444-9 (PMC9908950; doi:10.1038/s41598-023-29444-9)
Supplement: Supplementary file 1 — Supplementary Information. [file 41598_2023_29444_MOESM1_ESM.docx]

Spatial helicity response metric to quantify particle size and turbidity of heterogenous media through circular polarization imaging: supplemental document

# S1. Additional methods

## S1.1 Beam splitter Stokes-Mueller calculus

It must be noted that, upon reflection, the beam splitter transformed the two backscattered circular polarization states (right- and left-handed) from the sample into slightly elliptical states [1]; thus, R2 and P2 were set to transmit those elliptical states which corresponded to the right- and left-circularly polarized components of the sample’s backscattered light. To do this, the Mueller matrix of the beam splitter [1] was calculated as

| $\text{M}_{\text{BS, reflection}}\text{=}\left[ \begin{aligned} \text{1}\text{ } \\ \text{sin(2}\text{θ}_{\text{r}}\text{)}\text{ } \\ \text{0 } \\ \text{0 } \end{aligned}\begin{aligned} \text{sin(2}\text{θ}_{\text{r}}\text{)}\text{ } \\ \text{1}\text{ } \\ \text{0 } \\ \text{0 } \end{aligned}\begin{aligned} \text{0} \\ \text{0} \\ \text{ }-\text{cos(2}\text{θ}_{\text{r}}\text{)}\text{ } \\ \text{0} \end{aligned}\begin{aligned} \text{ 0} \\ \text{ 0} \\ \text{ 0} \\ \text{ }-\text{cos(2}\text{θ}_{\text{r}}\text{)} \end{aligned} \right]$. | (S1) |
| --- | --- |

The reflection angle $\text{θ}_{\text{r}}$ in Eq. (S1) is calculated using Snell’s law, with angle of incidence $\text{θ}_{i}=$ 45$^{\circ}$, and refractive indices for air $n_{\text{air}}=$ 1 and glass $n_{\text{air}}=$ 1.52:

| $\text{θ}_{\text{r}}=\text{sin}^{-1}\left( \frac{n_{\text{air}}}{n_{\text{glass}}} \text{sin}\text{θ}_{i} \right)$. | (S2) |
| --- | --- |

The transformation of the transmitted light due to the beam splitter was not taken into account since the ellipticity change was negligible (~5%).

## S1.2 Monte Carlo model

Our previously developed, validated, and publicly available polarization-sensitive Monte Carlo model [2–6] was employed to corroborate the experimental circular polarization images and provide additional insight through tracked photon statistics. The details of the model’s implementation can be found here [7].

The simulation parameters were set to match those of the experiment as best as possible. The light source launched ~10^6^ left-circularly polarized photon packets (λ $=$ 635 nm) into a 2.2×2.2×2.2 cm medium of monodispersed spheres, each with a refractive index of 1.59, suspended in a host refractive index of 1.33. The model tracked the position, direction, and polarization of each photon packet during their propagation in the medium. The average number of scattering events and average scattering angle per detected circularly polarized photon were tracked as well.

In the simulation, as each photon packet met a scatterer, its new direction was determined by sampling the theoretical scattering intensity distribution (i.e., phase function) with respect to angle, its polarization was transformed through single scattering Stokes-Mueller calculus (Eq. (1) and (2) in the primary manuscript), and its number of scattering events and average scattering angle were updated. The detector was a 400×400 bin element placed in the 180° backscattering direction to match the experimental configuration. The detector element binned each photon packet’s accompanying set of statistics based on the location of detection and then summed the statistics to yield 2D arrays of numerical results. A typical simulation took ~1 hour on a laptop PC with an Intel Core i5-7200U 2.71 GHz processor.

# S2. Supplementary figures


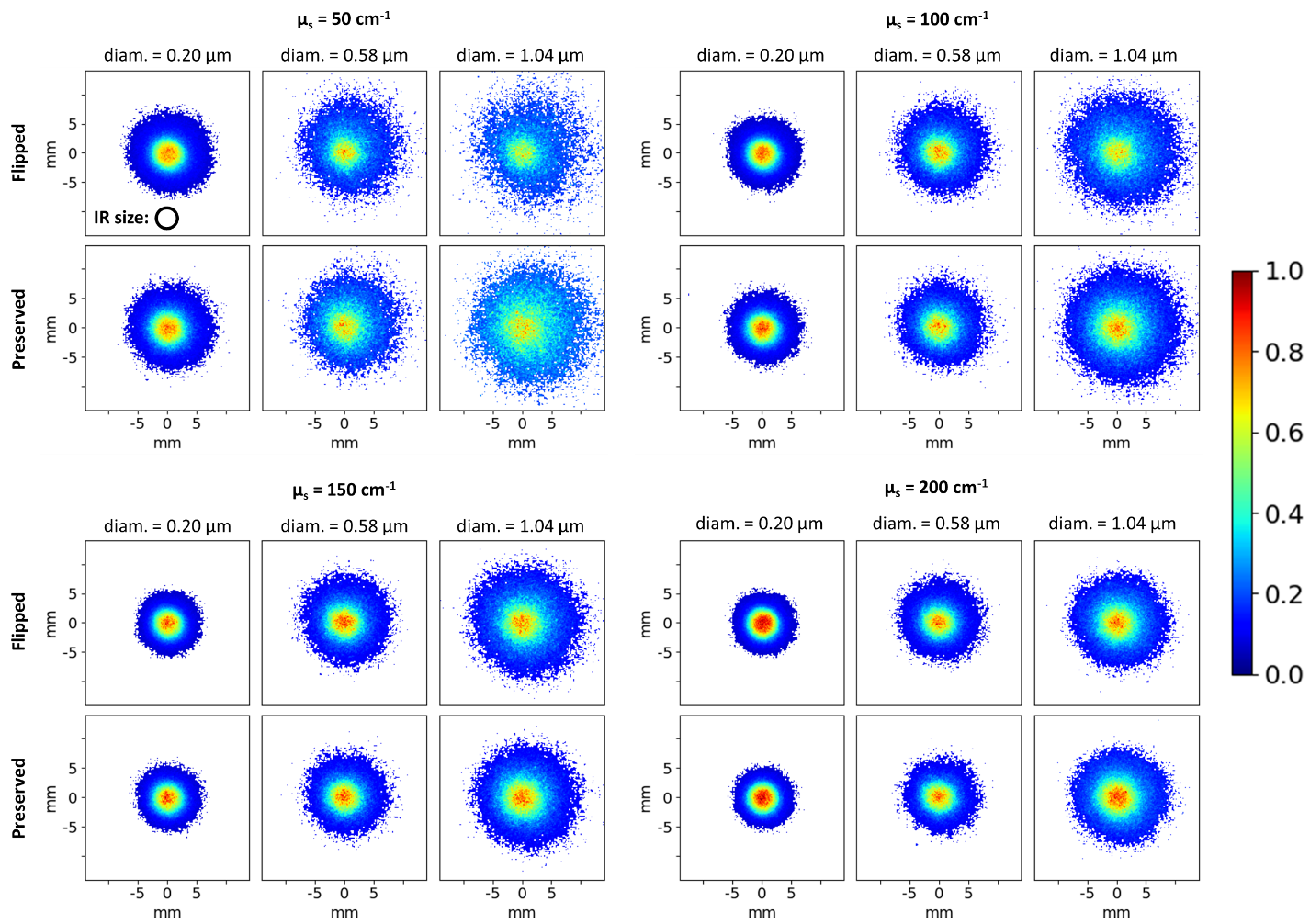


Fig. S1. The full set of experimental images. Helicity-flipped (top row of each panel) and helicity-preserved (bottom row of each panel) intensity images of backscattered circularly polarized light from twelve different monodispersed polystyrene suspensions, each having a sphere diameter of 0.20 μm, 0.58 μm, or 1.04 μm and scattering coefficient of 50 cm^‑1^, 100 cm^‑1^, 150 cm^‑1^, 200 cm^‑1^ (see labels). It is visually apparent that the characteristics of resultant images are dependent on the scatterer size and turbidity (for discussion of noted trends, see text). The dashed circle in the top-leftmost image demarcates the centrally illuminated region IR.


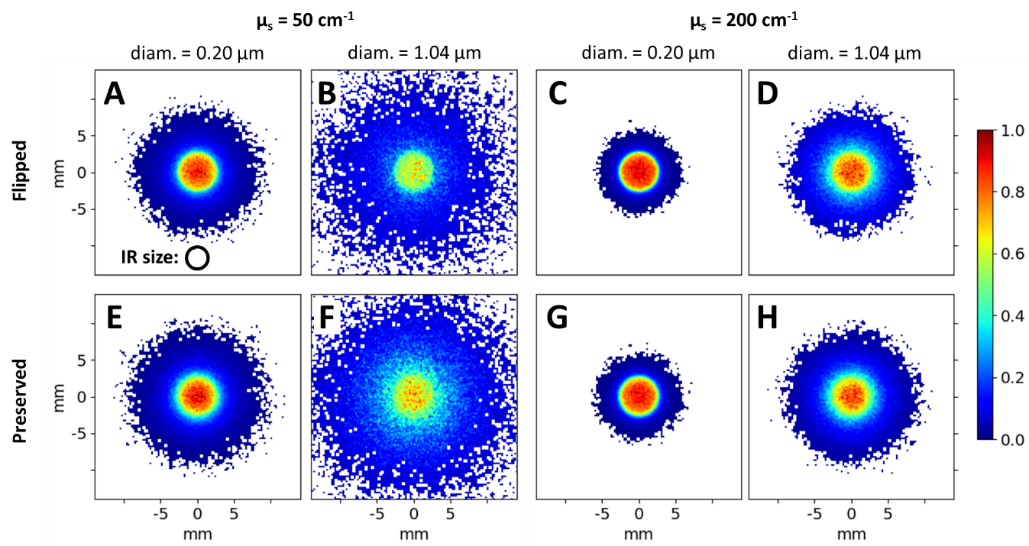


Fig. S2. Simulated versions of the images found in Figure 3 for validation; see its accompanying caption and text for details. Briefly, helicity-flipped (top row) and helicity-preserved (bottom row) intensity images are shown for four different simulated suspensions, each having a sphere diameter of either 0.20 μm or 1.04 μm and scattering coefficient of either 50 cm^-1^ and 200 cm^-1^ (see labels). The dashed circle in (A) image demarcates the centrally illuminated region IR.

# REferenceS

1. E. Collett, *Field Guide to Polarization* (SPIE, 2009).

2. D. Côté and I. A. Vitkin, "Pol-MC: a three dimensional polarization sensitive Monte Carlo implementation for light propagation in tissue," http://www.novajo.ca/ont-canc-inst-biophotonics.

3. M. F. G. Wood, X. Guo, and I. A. Vitkin, "Polarized light propagation in multiply scattering media exhibiting both linear birefringence and optical activity: Monte Carlo model and experimental methodology," J Biomed Opt **12**(1), 014029 (2007).

4. X. Guo, M. F. G. Wood, and I. A. Vitkin, "Stokes polarimetry in multiply scattering chiral media: Effects of experimental geometry," Appl Opt **46**(20), 4491–4500 (2007).

5. N. Ghosh, M. F. G. Wood, and I. A. Vitkin, "Mueller matrix decomposition for extraction of individual polarization parameters from complex turbid media exhibiting multiple scattering, optical activity, and linear birefringence," J Biomed Opt **13**(4), 044036 (2008).

6. M. D. Singh and I. A. Vitkin, "Discriminating turbid media by scatterer size and scattering coefficient using backscattered linearly and circularly polarized light," Biomed Opt Express **12**(11), 6831 (2021).

7. D. Cote and I. A. Vitkin, "Robust concentration determination of optically active molecules in turbid media with validated three-dimensional polarization sensitive Monte Carlo calculations," Opt Express **13**(1), 148 (2005).
